# Recognizing Late Onset Frontotemporal Dementia with the DAPHNE scale: A case report

**DOI:** 10.1590/1980-57642018dn12-010011

**Published:** 2018

**Authors:** Leonardo Tafarello Martins, Ivan Abdalla Teixeira, Jerson Laks, Valeska Marinho

**Affiliations:** 1 Center for Alzheimer's disease and Related Disorders, Institute of Psychiatry, Universidade Federal do Rio de Janeiro, RJ, Brazil; 2 MSc Student Center for Alzheimer's Disease and Related Disorders, Institute of Psychiatry, Universidade Federal do Rio de Janeiro, RJ, Brazil; 3 Associate Professor, Universidade do Estado do Rio de Janeiro, RJ, Brazil; 4 PhD. Center for Alzheimer's Disease and Related Disorders, Institute of Psychiatry, Universidade Federal do Rio de Janeiro, RJ, Brazil; 5 Invited Professor, Postgraduate Program in Translational Biomedicine, Universidade do Grande Rio (Biotrans - Unigranrio)

**Keywords:** frontotemporal dementia, late onset, scale, DAPHNE, bipolar disorder, demência frontotemporal, início tardio, escala, DAPHNE, transtorno bipolar

## Abstract

Frontotemporal dementias are classically described as early onset dementias with personality and behavioral changes, however, late onset forms can also be found. Considering the paucity of information about late onset behavioral variant frontotemporal dementia and its challenging diagnosis, we present a case report of an 85-year-old woman with behavioral changes and slow progression to dementia who was first diagnosed as having bipolar disorder and then Alzheimer's disease. The Daphne scale provided a structured means to improve clinical diagnosis, also supported by characteristic features on MRI and SPECT, while CSF biomarkers ruled out atypical Alzheimer's disease.

## RESUMO

As demências frontotemporais são classicamente descritas como demências de início precoce com mudanças de personalidade e comportamento, porém as formas de início tardio também podem ser encontradas. Considerando a escassez de informações sobre a demência frontotemporal - variante comportamental de início tardio e o diagnóstico desafiador, apresentamos um relato de caso de uma mulher de 85 anos com alterações comportamentais e progressão lenta para demência que foi diagnosticada pela primeira vez com transtorno bipolar e, em seguida, doença de Alzheimer. A escala DAPHNE foi utilizada permitindo a estruturação das características clínicas, aumentando a precisão do diagnóstico clínico, apoiado por características em RM e SPECT, enquanto os biomarcadores no líquor descartaram a doença de Alzheimer.

Frontotemporal dementias (FTD) are classically described as early onset dementias, however, late onset forms account for up to 40% of all cases,^1^ manifested by behavioral and personality changes and language disturbances.^2^ FTD includes three clinical presentations: behavioral variant frontotemporal dementia, semantic dementia, and progressive nonfluent aphasia. New consensus criteria establish that, for a diagnosis of 'possible' behavioral variant frontotemporal dementia (bvFTD), three out of six clinically discriminating features (disinhibition, apathy/inertia, loss of sympathy/empathy, perseverative/compulsive behaviors, hyperorality and dysexecutive neuropsychological profile) are required. 'Probable' bvFTD criteria include functional disability and characteristic neuroimaging, whereas bvFTD 'with definite frontotemporal lobar degeneration' requires histopathological confirmation or a pathogenic mutation.^2^

Due to the major changes in personality and behavior, bvFTD is frequently misdiagnosed as a primary psychiatric disorder.^3^ An accurate differential diagnosis between bvFTD and psychiatric disorders must be established given the dramatic differences in prognosis, therapeutic options, and family orientation.^3^ As bvFTD diagnosis relies mainly on its clinical features, a behavioral inventory can help differentiating it from other diseases. Many behavioral inventories have been used since the first diagnostic criteria for FTD were published, including the Frontal Behavioral Inventory (FBI),^4^ the Middelheim Frontality Score (MFS),^5^ and a recently proposed tool based on the new bvFTD criteria, called the DAPHNE scale.^6^

The DAPHNE (an acronym for Disinhibition, Apathy, Perseverations, Hyperorality, Personal Neglect and loss of Empathy) is a six-domain, ten-item scale designed as a semi-structured interview. The first five domains were proposed from bvFTD Rascovsky criteria and the last from the FBI. Each item can be scored on a five-point scale (none, very mild, mild, moderate, severe). The scale validation process was successful in differentiating bvFTD from non-bvFTD patients, as well as from Alzheimer's Disease, Progressive Supranuclear Palsy and Bipolar patients.^6^

Differences in FTD clinical presentation according to age of onset have also been described.^1^ An overall worse neuropsychological performance, impairment in memory and visuospatial function, as well as more symptoms of depression, apathy, and impulsiveness, have been described in early onset FTD.^1^ Late onset forms have not been extensively described and may be less frequently diagnosed on clinical grounds and often misdiagnosed as Alzheimer's disease.

Considering the paucity of information about late onset bvFTD, we present a case report of an 85-year-old woman with marked changes in behavior and slow progression over a decade to cognitive and functional impairment, proving non-responsive to many pharmacological therapies. The Daphne scale provided a structured means to improve clinical diagnosis, including the challenging differentiation between bipolar disorder and bvFTD. Support to confirm bvFTD diagnosis was obtained by using neuroimaging and cerebrospinal fluid biomarkers.

## CASE REPORT

We present the case of Mrs. G, an 85-year-old retired lawyer, without any previous history of psychiatric disorder, who began to present a dramatic behavioral change at the age of 75.

She had a previous medical history of hypertension and stage I breast cancer at 71years of age (considered cured after quadrantectomy, radiation therapy, and hormone blockade treatment), and a family history of bipolar disorder (her older sister).

Contrasting with her previous parsimonious personality and good financial organization, she started spending excessively, buying jewelry and expensive clothes, lending money to others indiscriminately, and taking out bank loans. Also, although she had always been homosexual, she started to offer sex to unknown men in the neighborhood, manifesting hypersexuality and masturbating herself in front of others. Simultaneously, her speech became loud and fast, and she claimed feeling a lot of energy to do several activities at the same time. Later, she presented persecutory delusions, accompanied by auditory hallucinations, in which she believed that her neighbors were plotting to kill her. Over the ensuing months, she had sudden mood swings, cycling from euphoria to periods of great hopelessness, apathy, and tearfulness. These changes often occurred from one day to the next or even during the same day. At this point, she had no memory, language, or visuospatial disturbances. She was first seen by a psychiatrist and diagnosed as having bipolar mood disorder. There was no response or reasonable symptom control following any of the treatments prescribed, despite use of an extensive list of psychiatric medicines, including antipsychotics (ziprasidone, olanzapine, quetiapine, risperidone, thioridazine, periciazine, haloperidol, chlorpromazine, paliperidone, clozapine), mood stabilizers (lamotrigine, lithium, divalproex), benzodiazepines (alprazolam, bromazepam, diazepam, clonazepam, chlordiazepoxide) and antidepressants (sertraline, paroxetine, citalopram, trazodone, fluoxetine, venlafaxine, duloxetine, clomipramine, nortriptyline, amitriptyline, mirtazapine), prescribed either as monotherapy or combined therapy.

At the age of 80 years, her mood became predominantly sad, anxious, and apathetic, she developed stereotypical behaviors and compulsions of self-harm (such as biting and beating herself, nail-biting and hair-pulling), and repeating words or phrases out of context. When upset, she either threatened to commit suicide or displayed catastrophic reactions and voluntary falls. She started to complain of forgetfulness, with limited self-orientation outside the home and progressive loss of autonomy, remaining more restricted to the domestic environment. She gradually developed dependency for instrumental and basic activities of daily living, needing help with her self-hygiene, choosing clothes and dressing, feeding herself and handling money. Throughout the course of the disease, Mrs. G's sleep remained preserved with the use of benzodiazepines and there was no significant weight change. Often, she did not recognize caregivers or relatives, mistaking them as thieves. An AD diagnosis was suggested by another psychiatrist, who then prescribed rivastigmine and memantine. After this prescription, Mrs. G. showed a marked worsening in her behavior.

At 85 years old, she came to our service for an evaluation, brought by her partner, because of extreme self-mutilation, with bruises all over her body and major cognitive impairment. At that time, she was taking paroxetine 40 mg per day and clonazepam 2 mg per day. She presented with puerile and disinhibited behavior, labile affect, impoverished and perseverative thought, verbal and motor stereotypies, as well as primitive reflexes of frontal lobe release (glabellar and suction). At initial cognitive assessment, she scored 16/30 on the Mini-Mental State Examination, and 7/30 on the Montreal Cognitive Assessment (MoCA), revealing impairments in executive function, verbal fluency, and memory on broader neuropsychological testing. The functional impairment in activities of daily living, such as ability to use the telephone, shopping, or housekeeping, was assessed with the Instrumental Activities of Daily Living Scale,^7^ scoring 14/27. Mrs. G was considered dependent for dressing and toileting categories of the Katz Index, scoring 2/6.^8^ In order to best assess the clinical suspicion of frontal lobe degeneration syndrome, three specific scales were chosen: the Frontal Behavioral Inventory,^4^ the Middelheim Frontality Score,^5^ and the DAPHNE.^6^ The patient attained a score indicating possible bvFTD on all of these instruments (54/72 on FBI; 10/10 on MFS and 25/40 on DAPHNE). On the short screening version, DAPHNE-6, the patient scored 6/6, which is also above the suggested cut-off score of 4/6. DAPHNE scores were classified as 'severe' on items including unrestrained spending habits, sexual disinhibition, and personal neglect; 'mild' or 'moderate' in loss of social convenience, loss of initiative/social interest, emotional blunting/indifference, and perseverations/fixed ideas/stereotypical behavior and as 'very mild' for eating disorders (gluttony and joviality were absent).

**Figure 1:**
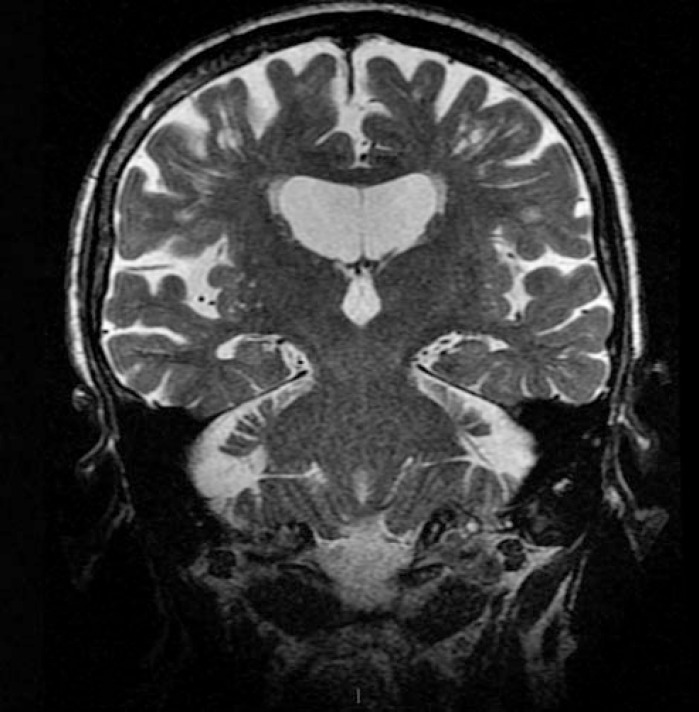
MRI with difuse cortical atrophy, frontal predominance.

**Figure 2:**
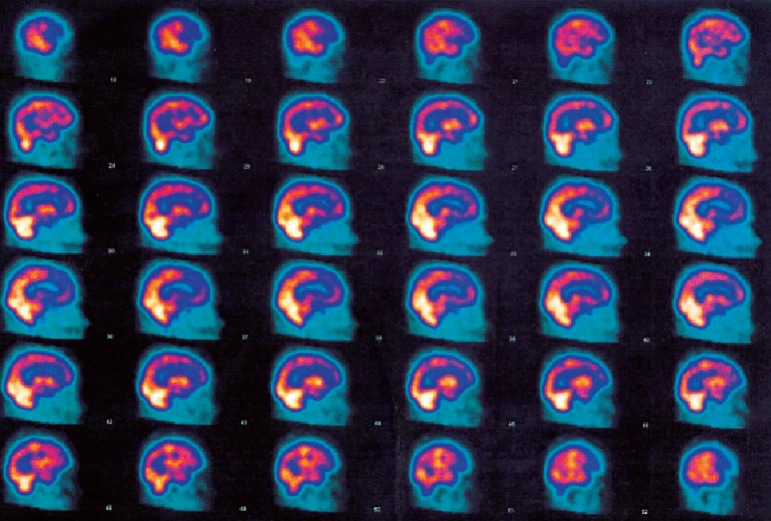
SPECT with Frontal lobes hypoperfusion.

Recent magnetic resonance imaging revealed diffuse cortical atrophy, with frontal predominance; hippocampi were only slightly reduced, with a score of 1 on the Scheltens scale^9^ (Figure 1). In order to further confirm the diagnosis of frontotemporal lobe syndrome, single-photon emission computed tomography (SPECT) was ordered, which showed preferred hypoperfusion of the frontal lobes (Figure 2). In order to exclude atypical frontotemporal presentation of AD, lumbar puncture was performed. CSF biomarkers beta-amyloid 42, tau and phospho-tau, were all within the normal range (674 pg/ml; 209 pg/ml; 22.2 pg/ml; respectively).

After excluding the diagnosis of AD on the grounds of the CSF biomarkers profile, a diagnosis of probable bvFTD was established. We then proposed discontinuation of all medications and the introduction of trazodone as a monotherapy. Mrs. G showed a partial improvement of the behavioral symptoms with trazodone, but not of the functional and cognitive impairments, and so the dose was escalated to 300 mg/day after two months of titration.

## DISCUSSION

The present report describes a late-onset bvFTD case with slow progression to functional and cognitive decline, initially misdiagnosed as a primary psychiatric disorder. This is in line with current data showing that persons with bvFTD receive a psychiatric diagnosis more frequently than patients with AD (50.7% vs 23.1%).^10^ Common bvFTD psychiatric symptoms, such as anhedonia, apathy, mood swings, disinhibition, and repetitive and stereotyped behaviors, as well as psychotic symptoms, frequently lead clinicians to a misdiagnosis of bipolar disorder, depressive disorder, obsessive compulsive disorder, or schizophrenia.^3^ Late-life bipolar disorder, although less common than in younger ages, should be recognized as a possible differential diagnosis. Also, as behavioral changes and functional decline may be seen in some psychiatric conditions, a precise diagnosis may be a challenge.

The current bvFTD diagnostic criteria^2^ suggest that if behavioral changes are best explained by a psychiatric disorder, the diagnosis of FTD should be postponed. Nevertheless, only a minority of patients with probable and possible bvFTD diagnosis can be classified in any category of mental disorder, if formal criteria of mental disorder, such as those used in DSM IV and ICD-10, are applied to these cases.^11^

Some "red flags" are suggested to raise the suspicion of a diagnosis of bvFTD, including prominent cognitive impairment, progressive functional decline, lack of response to psychiatric treatment, late-onset and/or long-lasting manic or sustained depressive states (not episodic), and late-onset psychotic disorders spectrum.^3^ All of these reported red flags were also found in the present report, making bipolar disorder a less likely diagnosis.

The average survival time in FTD is 8.7 years^12^ and both cognitive and behavioral symptoms have a poor treatment response.^3^ An early correct FTD diagnosis is important to help families to provide overall better caregiving and to avoid distressing misinformation,^3^ where information about diagnosis and psychosocial support through educated staff may help reduce caregiver burden. Indeed, it has been reported that caregivers of persons with bvFTD are usually more distressed by profound changes in the patient's behavior and in interpersonal relationships.^13^

In addition to psychiatric conditions, other neurodegenerative diseases such as Lewy bodies dementia, vascular dementia, corticobasal degeneration, progressive supranuclear palsy, Huntington's disease, and AD should be considered in the differential diagnosis among patients with late onset behavioral changes. A behavioral inventory can provide valuable information for identifying behavioral symptoms, contributing to an accurate diagnosis of bvFTD and also helping to differentiate bvFTD from other diseases. Such inventories can be sensitive tools in FTD assessment, but many of them were found to be based on previous diagnostic criteria or were not validated for pure psychiatric disturbances.^14^

The DAPHNE scale is a recently developed tool, adapted from the new revised criteria by Rascovsky, and has demonstrated good psychometric properties. The simplified version (DAPHNE-6) can be used as a screening tool with 92% sensitivity (4/6 cut-off), whereas the complete version (DAPHNE-40) can be used for differentiating bvFTD from other conditions, including other degenerative diseases and pure psychiatric disorders such as BD, (15/40 cut-off) with a specificity of 92%.^6^ In the present report, we chose DAPHNE as the main screening and diagnostic instrument, since mood and behavioral disturbances, as well as family history of bipolar disorder, prompted an initial diagnosis of BD.

Nevertheless, focal cortical presentations of AD with predominant behavioral symptoms can account for some diagnostic issues.^15^ Post-mortem studies have shown that about 10-40% of patients with clinical diagnosis of bvFTD present AD pathology.^16^ Unlike bvFTD, even the behavioral variant of AD usually shows early memory loss, and CSF biomarkers, such as the amyloid protein and tau, can provide valuable information on the distinction between bvFTD and atypical presentations of AD.^11^ Biomarkers have been included in the current diagnostic criteria of bvFTD,^2^ and are considered an important tool in clinical settings.

It was recently demonstrated that MRI, FDG-PET, and CSF biomarkers can help guide a precise differential diagnosis between bvFTD, psychiatric disorders, and other neurodegenerative disorders.^17^ While FTD presents with hypofunction/hypoperfusion or even with marked atrophy in frontal and temporal lobes from the very onset of the disease, AD presents predominant hypofunction/hypoperfusion or atrophy in hippocampi and parietal lobes. Some forms of FTD can have nonspecific increased tau and phospho-tau levels in CSF, but beta amyloid protein generally remains within the normal range, distinguishing the typical AD signature, with low beta amyloid CSF concentration.^18^ Primary mental disorders do not usually present marked changes in neuroimaging or CSF biomarkers.

In the case reported, despite the major change in Mrs. G's personality, the correct diagnosis brought comfort to the family and to the patient, as it enabled them to understand the reason for the progressive course of the disease and the worsening in cognition and functional activities. It also allowed the family to organize a better daily routine to address Mrs. G's needs. In addition, the introduction of trazodone, a drug with cumulative evidence in the treatment of FTD,^19^ showed some effect in controlling the behavioral symptoms, improving the patient's quality of life and reducing the caregivers' distress. There is also consistent literature pointing to a worsening of behavioral symptoms in bvFTD with the use of cholinesterase inhibitors, as occurred in the case described.^20^

In conclusion, late onset bvFTD can pose a major diagnostic challenge. A precise clinical evaluation should take into consideration the new diagnostic criteria and the use of behavioral inventories, neuroimaging and CSF biomarkers to establish an accurate diagnosis. Further data on the clinical characteristics and course of late-onset bvFTD are needed to help clinicians establish an earlier and more precise diagnosis, which contributes to the organization of better care of patients and helps reduce caregiver distress.
